# Treatment of hepatitis C virus genotype 4 in the DAA era

**DOI:** 10.1186/s12985-018-1094-4

**Published:** 2018-11-22

**Authors:** Antonio Di Biagio, Lucia Taramasso, Giovanni Cenderello

**Affiliations:** 1 Infectious Diseases Clinic, Policlinico Hospital San Martino, L.go R. Benzi n 10, 16132 Genoa, Italy; 2 Department of Health Sciences (DISSAL), University of Genova and Infectious Disease Clinic, Via Pastore n 1, 16136 Genoa, Italy; 3 Infectious Diseases Unit, Department of Internal Medicine, ‘Fondazione IRCCS Ca’ Granda Ospedale Maggiore Policlinico, Via Francesco Sforza 35, Milan, Italy; 4 Infectious Disease Unit, EO Ospedali Galliera, via Mura delle Cappuccine n 14, 16128 Genoa, Italy

## Abstract

The recently approved interferon-free DAA (direct antiviral agents) regimens have shown not only to be effective in terms of sustained virological response (SVR) rates (> 90%) but also well tolerated in most hepatitis C virus (HCV) infected patients. Nevertheless HCV genotypes are different and only a small percentage of trials consider genotype 4 (GT4), which was associated with lower rates of SVR compared with other genotypes before the arrival of the DAA’s. In this review, we discuss the efficacy of DAA therapy in GT4 HCV infection with specific reference to more recent studies, including those conducted in a ‘field-practice’ scenario. Overall, DAA-based regimens appear more effective also in the poorly-explored setting of patients with HCV GT4 infection. Despite an overall limited number of patients was evaluated, favorable results are being derived from studies on ombitasvir/paritaprevir/ritonavir, sofosbuvir and velpatasvir, whether or not in association with voxilaprevir, and with the new combined therapy glecaprevir + pibentasvir.

## Introduction

The introduction of direct antiviral agents (DAAs) in clinical practice has undoubtedly represented a landmark advance in the treatment of hepatitis C virus infection (HCV) [1, 2]. With these therapies, the wide majority (90–95%) of HCV-infected patients can now be successfully cured [2, 3]. Less attention was paid to genotype 4 (GT4) HCV in clinical trials and field-practice studies compared with other genotypes [4]. Current estimates state that this GT accounts for 13% of all HCV infections [5]. The bulk of the GT4 infection is found in the Middle East, Northern Africa and Sub-Saharan Africa, with 93% of HCV patients in Egypt being infected by this genotype [5, 6]. In Europe, prevalence of GT4 infection is more variable (up to 16% in Italy in HIV co-infected patients, 8% in Spain, 14% in Belgium); however, migration flows from Northern Africa are likely to increase the prevalence of GT4 HCV infection in the next years [5, 7]. Importantly, GT4 is a historically difficult to treat genotype, and a trend towards lower rates of sustained virological response (SVR) in GT4 has been signaled also with DAA treatment [1]. A recent excellent review by Hathorn and Elsharkawy has extensively discussed current evidence on the treatment of GT4 HCV infection in the DAA era, with a focus on clinical trials [4]. However, the advancement of clinical research in HCV treatments proceeds at such a high pace that continuous update is necessary [4]. In particular, information on patients with renal disease - who often present HCV infection and who are particularly challenging to treat- appears required.

In this review, we discuss the efficacy of DAA therapy in GT4 HCV infection with specific reference to more recent studies, including those conducted in a ‘field-practice’ scenario.

## Methods

Papers for consideration in the present review were retrieved via a PubMed query, using “DAA AND HCV AND genotype 4” as keywords. Research was last updated on 22nd Sept 2017. No other limitations were applied. In total, 247 papers met these criteria. Papers were then selected for inclusion in the present manuscript according to their relevance for the topic, as judged by the Authors. When possible, studies with specific reference to GT4 infection were selected. Overall, 17 clinical trials (one in patients undergoing hemodialysis), 17 ‘field-practice’ experiences (two of which on patients with renal disease) and one meta-analysis of real-world data were reported [Table 1].

**Table 1:** Key results of the clinical trials included in the present analysis

Clinical trial or field experience	n° of patients with G4-infection	Treatment	SVR rate in GT4 patients
Ruane 2015 [8]	34	SOF + RBV	68–93%
Feld 2015 [9]	116	SOF/VEL	100%
Curry 2015 [10]	8	SOF/VEL	100%
Gane 2016 [11]	17	SOF/VEL + VOX	58%
Bourliere 2017 [12]	41 (POLARIS-1 + POLARIS-4)	SOF/VEL + VOX	97%
Colombo 2017 [13]	10	SOF/LDV	100%
Kholi 2015 [14]	21	SOF/LDV	95%
Buti 2017 [15]	40	SMV/SOF	100%
El Raziky 2017 [16]	63	SMV/SOF	92%
Kwo 2017 [17]	37	EBR + GZR	89%
Waked [18]	160	OBV/PTV/R/RBV	93–94%
Hézode 2015 [19]	135	OBV/PTV/r ± RBV	100% with RBV, 91% without RBV
Forns [20]	16	GLI/PIB	100%
Kwo [42]	22(SURVEYOR-I and SURVEYOR-II)	GLI/ PIB	100%
Asselah [21]	SURVEYOR-II Part 4, ENDURANCE-4 and ENDURANCE-2)	GLI/ PIB	93% 8 week treatment 99% 12 week treatment
Yakoot [22]	120 (randomized 60 in 12 weeks regimen and 60 in response tailored regimen)	SOF + DAC	96.7% in the fixed regimen 98.4% in the response tailored regimen
El-Khayat [23]	551 cirrotic patients (432 naïve, 119 treatment experienced)	SOF + DAC + RBV	92%in naïve 87% in experienced
Willemse [24]	53 (naïve and IFN experienced)	SOF + SIM	92%
Degre [25]	87, IFN experienced	SOF + SIM ± RBV	87.4%
Elsharkawy 2017 [26]	8742 (F3) 5667 (F3)	SOF/peg/IFN-RBV SOF/RBV	94% 79%
Asselah 2017 [27]	67 naïve, F0-F2	SIM + PEG IFN	97%
Ioannou [28]	135 (two arm: 104, 31)	SOF/LDV ± RBV and OBV/PTV/r ± RBV	89.6%
Crespo [29]	152 (two arm:130 and 122)	SOF/LDV ± RBV and OBV/PTV/r ± RBV	95.4 and 96.2%
Welzel [30]	53	OBV/PTV/r ± RBV	100%
Perello [31]	87 (73% cirrhosis, 35% treatment naïve)	OBV/PTV/r ± RBV	100%
Petta 2017 [32]	17	OBV/PTV/r ± RBV	94%
Wedemeyer 2017 [33]	112, 19 cirrhosis	OBV/PTV/r ± RBV	96,5%
Komatsu [34]	26	EBR + GZR	100%
Maria 2017 [35]	7	SOF ± SIM or DAC or LDV ± RBV	100%
Hezode 2016 [36]	215	SOF + DAC	91%
Babatin 2017 [37]	40 35% cirrhotic; treatment-experienced 52.5%	SOF + DAC + RBV	100%
Abad 2017 [38]	35	OBV/PTV/r	100%
Ponziani 2017 [46]	8	OBV/PTV/r	100%
Manns 2016 [43]	37	SOF/LDV + RBV	81%

*GT4* HCV Genotype 4, *SOF* sofosbuvir, *VEL* velpatasvir, *VOX* voxilaprevir, *LDV* ledipasvir, *SIM* simeprevir, *RBV* ribavirin, *EBR* elbasvir, *GZR* grazoprevir, *OBV* ombitasvir, *PTV* paritaprevir, *GLI* glicaprevir, *PIB* pibrentasvir, *DAC* daclatasvir

### Clinical trials

#### Sofosbuvir

In an open-label study at single center, 60 patients were enrolled and completed treatment with Sofosbuvir plus ribavirin for 12 weeks (31 patients) or 24 weeks (29 patients). SVR 12 was achieved by 21 of the 31 patients (68%) receiving 12 weeks of treatment, and by 27 of 29 patients (93%) receiving 24 weeks of treatment [8].

#### Sofosbuvir/velpatasvir

In clinical trials, the association of sofosbuvir and velpatasvir (SOF/VEL) showed promising results. In a phase 3, double-blind, placebo-controlled study, 116 patients with GT4 infection (52/116 treatment experienced, 27/116 cirrhotic) were treated with such association, reaching 100% SVR [9]. The same association was also investigated in 267 patients with decompensated cirrhosis in the ASTRAL-4 study [10]. In this difficult-to-treat population, serious adverse events occurred in 19% of patients who received 12 weeks of SOF/VEL, 16% of those who received 12 weeks of SOF/VEL plus ribavirin (RBV), and 18% of those who received 24 weeks of SOF/VEL. Only eight patients infected with GT4 have been included in the study and all achieved SVR.

#### Sofosbuvir/velpatasvir/voxilaprevir

In a multicenter, phase II study on sofosbuvir, velpatasvir and voxilaprevir (SOF/VEL/VOX), Gane et al. have investigated the optimal regimen for patients with GT1, 2, 3, 4 or 6 infections [11]. In total, 128 patients were enrolled; of these, 17 presented GT4 infection (10 treatment-naïve and 7 with prior therapy). In total, only 7 patients experienced a virological failure, of whom 3 were GT4 infected. All three patients were treatment-naïve, two were non-cirrhotic patients treated for 6 weeks, while the third was a cirrhotic patient treated for 8 weeks. Additional data come from the POLARIS-1 and POLARIS-4 studies, in which 22 and 19 patients infected with HCV GT4 have been included, respectively, and were treated with SOF/VEL/VOX [12]. All patients had been previously treated with a DAA-containing regimen; 21/22 subjects in POLARIS-1 and 19/19 in POLARIS-4 achieved SVR. The only treatment failure, in POLARIS-1, occurred in a cirrhotic patient who had been previously treated with SOF and ledipasvir (SOF/LDV) for 12 weeks in 2015, while no other patient with GT4 experienced treatment failure with this triple drug regimen.

#### Sofosbuvir/ledipasvir

In an international, randomized, phase II, open-label study, the safety and efficacy of SOF/LDV in fixed-dose combination tablet, for 12 or 24 weeks were compared in 114 kidney transplant recipients (69% treatment-naïve) with GT1 (91%) or GT4 infection (9%) [13]. Remarkably, all patients, regardless of treatment duration, achieved SVR12. Serious adverse events were reported in 13 patients (11%), and one patient permanently discontinued treatment due to an adverse event (syncope). In a proof-of-concept, single-center, open-label, phase 2a trial, SOF/LDV was also studied exclusively in patients infected with GT4 [14]. In this study, only 21 patients have been included, but 20 of them achieved SVR after 12 weeks of SOF/LDV (95%), including seven patients with cirrhosis, while the only patient who failed achieving SVR was non-adherent to treatment. No patients discontinued therapy due to adverse events and no grade 3 or 4 adverse events related to study medications were reported.

#### Simeprevir

Two recently published clinical trials investigated the efficacy of simeprevir (SMV) + SOF in GT4 patients: PLUTO and OSIRIS studies [15, 16]. The PLUTO study is a phase III open-label trial, that investigated the efficacy of 12-weeks treatment with SMV + SOF in GT4-infected patients either naïve or experienced to antiviral therapy [15]. In total, 40 patients were evaluated; of these, 27 (68%) were treatment-experienced and 7 (18%) had compensated cirrhosis. Noteworthy, all patients achieved SVR12, while adverse events, all of mild-to-moderate intensity, were reported in 20/40 (50%) of patients. The other recently published trial evaluating the efficacy and safety of SMV + SOF was the OSIRIS study, in which GT4 HCV-infected patients with F0-F4 fibrosis were treated for 8 or 12 weeks [16]. In total, 63 patients (33 treatment-naïve and 30 Peg-IFN/RBV-experienced) were randomly assigned to receive either 8 weeks (Group A1, *n* = 20) or 12 weeks (Group A2, n = 20) of treatment. Patients with compensated cirrhosis (METAVIR F4) received 12 weeks of treatment (Group B, *n* = 23). Overall, 92% of patients achieved SVR12; corresponding figures were 75% in Group A1 and 100% in both groups A2 and B. The five patients who did not achieve SVR12 experienced viral relapse during the first 32 days following treatment and were all prior Peg-IFN/RBV null responders. No patients discontinued due to treatment-emergent adverse events.

#### Elbasvir and grazoprevir

In a phase III, randomized, controlled, open-label trial, the effects of 12 or 16 weeks of treatment with elbasvir (EBR) and grazoprevir (GZR) in fixed-dose combination tablet, with or without twice-daily RBV, were evaluated in 420 treatment-experienced patients with GT1, GT4 or GT6 infection [17]. Patients were randomly assigned (1:1:1:1) to treatment with EBR + GZR once daily, with or without twice-daily RBV, for 12 or 16 weeks. Thirty-seven patients were GT4 (47% cirrhotic). Of them, 24 received 12 week-treatment (9 without and 15 with RBV) and 13 were treated for 16 weeks (5 without and 8 with RBV). The global proportion of SVR12 in patients infected with GT4 was 88.9%, with the highest rate of failure, 12.5%, in patients treated for 12 weeks without RBV and the lowest, 0%, in patients treated for 16 weeks with RBV.

#### Ombitasvir/paritaprevir/ritonavir

The efficacy of OBV/PTV/r in GT4 have also been explored in a multicentre phase 2b, randomised, open-label combination trial (PEARL-I), in which 135 patients with GT4 (49/135 treatment experienced) have been included and randomly assigned to receive once-daily OBV/PTV/r for 12 weeks with or without RBV [18]. SVR was achieved by 100% of patients treated with the addition of RBV and by 91% of those treated without. Only three treatment-naive patients who received OBV/PTV/r without RBV experienced virological failure, and they were all infected with GT4. One patient in OBV/PTV/r experienced an aspartate aminotranferase (AST) elevation of grade ≥ 3 and three patients in OBV/PTV/r + RBV experienced a bilirubin elevation of grade 3.

In the AGATHE-II a phase 3, open label, 182 patients with GT4 were screened, of whom 160 were eligible for inclusion; SVR was achieved by 94% (94/100) of patients without cirrhosis, and 97% (30/31) patients with cirrhosis treated for 24 weeks; Instead SVR was achieved by 93% (27/29) of patients with cirrhosis treated for 24 weeks [19].

#### Glecaprevir/pibrentasvir

The recently approved once-daily, ribavirin-free, DAA regimen of glecaprevir co-formulated with pibrentasvir, has been studied for pangenotypic HCV treatment. In the phase 3 study EXPEDITION-1, 16 GT4 infected patients with compensated cirrhosis had 100% sustained response after 12 weeks of treatment [20], and the same (100% SVR) was also found in SURVEYOR-I and SURVEYOR-II phase II, dose-ranging trials, despite the presence of baseline polymorphisms, in 22 GT4 infected patients [20]. In 3 separate phase 3 trials performed in patients without cirrhosis, (SURVEYOR-II Part 4, ENDURANCE-4 and ENDURANCE-2), 43/46 GT4 patients treated for 8 weeks and 75/76 patients treated for 12 weeks achieved SVR [21].

#### Daclatasvir

A recent paper reports a successful experience in treating patients with GT4 chronic infection with generic formulations of SOF and daclatasvir (DAC) [22]; in this open-label randomized non-inferiority study, in which all enrolled patients were non-cirrhotic and had GT4 chronic infection, two different schedules of treatment were compared. Patients were divided into two groups: a fixed duration group, in which all patients received 12 weeks of therapy, and a virological guided response group, in which patients could receive either 8 or 12 weeks of therapy, based on a virological response. Indeed, patients were treated for 8 weeks only if they achieved a very rapid virological response (vRVR), defined as an undetectable viral load after 2 weeks of treatment (patients not reaching vRVR were continuing treatment at 12 weeks). This trial showed a SVR12 of 96.67% (ITT) in the group with a fixed duration and of 98.33% in the virological guided response group. No major adverse events were observed, while a shorter treatment period could increase adherence, reduce the drug exposure and optimize resource use, reducing the cost of about one third [23]. A second multicentre, open-label trial, conducted exclusively in GT4 patients, describes the experience with SOF and DAC in patients with more advanced stages of disease. Five hundred fifty-one cirrhotic patients (468 Child A; 83 Child B) were enrolled to receive SOF and DAC plus weight-adjusted RBV for 12 weeks. Among them, 432 were naïve to IFN-treatment, whereas 119 were treatment-experienced. In the intention to treat analysis, the overall 12 week SVR rate was 92% in naïve patients, but lower, 68%, in patients presenting more than one negative predictor of response (treatment experienced and Child B cirrhotic). The corresponding figures in per protocol analysis were 94 and 79%. [23].

### ‘Field-practice’ experiences

#### Simeprevir

In a multicenter, ‘field-practice’, retrospective study, Willemse et al. have specifically assessed the effectiveness of treatment with SOF and SMV, with or without RBV, in a cohort of GT4 HCV patients [24]. A total of 53 patients, either naïve or experienced, were included. SVR rate was 92% (49/53). All four failures showed virological relapse and were not treated with RBV. In another experience, conducted in Belgium, Degrè et al. evaluated 87 GT4-infected treatment-naïve or IFN-experienced patients treated with SOF and SMV with or without RBV (41% had severe fibrosis, and 59% cirrhosis) [25]. The overall SVR12 rate was 87.4%, while patients treated with and without RBV had rates of 87.9 and 87%, respectively. On the other hand, patients with advanced fibrosis and patients with cirrhosis had SVR12 rates of 94.4 and 82.4%, respectively. SVR12 rates in treatment-naïve patients and in INF-experienced patients were 78.9 and 89.7%. In most cases, treatment failure occurred in patients with cirrhosis and severe disease. The treatment was well tolerated and no patient discontinued treatment due to adverse events.

In a multicenter study, Asselah et al. investigated the efficacy and safety of 12-week SMV plus peg-IFN/RBV in 67 treatment-naïve patients with GT4 infection and F0-F2 fibrosis [26]. Patients with early virologic response (HCV RNA < 25 IU/mL) at Week 2 and HCV RNA undetectable at weeks 4 and 8 were eligible to stop all treatment at the end of week 12 (*n* = 34), otherwise Peg-IFN/RBV therapy was continued to week 24. All patients in the 12-week group showed undetectable HCV RNA at end of treatment, and 97% (33/34) achieved SVR12. No new safety signals were reported.

#### Sofosbuvir

*A large National Treatment Programme was launched in Egypt practice in October 2014. Patients who were eligible for treatment were classified according to their eligibility for interferon therapy: Group 1 were treated with triple therapy for 12 weeks and Group 2 (interferon ineligible) were treated with Sofosbuvir and ribavirin for 24 weeks. The study analysed the data from the first 14,409 patients who completed follow-up to 12 weeks post HCV treatment. SVR12 rates were 94% and 78.7 in Group 1 and Group 2, respectively* [27]*.*

#### Sofosbuvir/ledipasvir

A large ‘field-practice’ retrospective analysis (*N* = 17,487) of the Veterans Affairs National Health Care System has evaluated the effectiveness of SOF/LDV and OBV/PTV/r in the treatment of 135 patients with HCV GT4 infection [28]. Of them, 104 received SOF/LDV ± RBV and 31 OBV/PTV/r ± RBV. Overall, SVR12 rate was 89.6% (95% CI 82.8–93.9), with a higher incidence in treatment-experienced subjects (93.5%). Interestingly, 96.4% of GT4-infected, OBV/PTV/r-treated patients achieved SVR12, compared with 87.6% of those receiving SOF/LDV. A smaller, but prospective, Spanish cohort study confirmed the above-mentioned findings [29]. In more details, GT4-infected patients treated with OBV/PTV/r + RBV (*n* = 122) or SOF/LDV ± RBV (*n* = 130) were evaluated. SVR12 rates were 96.2% with OBV/PTV/r + RBV and 95.4% with SOF/LDV ± RBV. In cirrhotic patients, SVR12 was 91.2% with OBV/PTV/r + RBV and 93.2% with SOF/LDV ± RBV. Rates of severe adverse events and associated discontinuations were 5.7 and 2.5% in the OBV/PTV/r sub-cohort, respectively and 4.6 and 0.8% in the SOF/LDV sub-cohort, respectively; in most cases, these events were likely due to RBV administration (e.g., anemia).

#### Ombitasvir/paritaprevir/ritonavir

In addition to the above-mentioned experiences (see previous paragraph, [25–28]), in another ‘field-practice’ study, from the German Hepatitis C Registry, Welzel et al. investigated the effectiveness of OBV/PTV/r ± RBV in 53 patients infected by GT4: all of them achieved SVR12 [29]. Similar findings were reported in a Spanish early access program, the effectiveness and safety of OBV/PTV/r was retrospectively investigated [30]. In total, 291, either infected 69.8% by GT1 or 30.2% by GT4 (73% with cirrhosis, 35% treatment-naïve) were evaluated. The rate of SVR12 was 96.2% for GT1 and 100% for GT4, with no statistical difference according to viral genotype. Thirty patients (10.3%) experienced serious adverse events, leading to discontinuation in six cases, once again due in most cases to RBV treatment. Although the number of GT4 patients was small, a prospective observational study, with collected data from ABACUS compassionate-use program, has investigated the use of OBV/PTV/r with dasabuvir plus RBV for GT1 and OBV/PTV/r plus RBV for GT4 infection (24 weeks of treatment) in patients with cirrhosis at high risk of decompensation, while approval of these regimens was pending in Italy [31]. In total, 762 patients were evaluated. Of them, only 17 had GT4 and 16 (94%) achieved SVR. The number of patients infected by GT4 was too low to allow a sub-group analysis, but, in the global population of this study, logistic regression analyses identified that bilirubin concentrations of less than 2 mg/dL were associated with SVR12 (odds ratio [OR] 4.76 [95% CI 1.83–12.3]; *p* = 0·001. Overall, the SVR rate was 96%; 23% of patients experienced adverse events, with asthenia being the most commonly reported (5%).

A meta-analysis of real world data of a total of 5158 patients (5046 with HCV GT1 and 112 GT4 infection), treated with OBV/PTV/r ± DSV ± RBV across 25 primary publications, showed an overall SVR of 96.8% (95% CI, 96.3 97.3) [32]. Cohort data included fewer patients with GT4 infection and cirrhosis, and breakdown data was available for 19 of them; 99% (95%CI, 82.3–100) of them achieved SVR. The rate of response was similar for non-cirrhotic patients with GT4 infection, as 100% (51/51) achieved SVR.

#### Elbasvir/gazoprevir

The independent analysis of the US Food and Drug Administration has shown that 100% (26 out of 26) patients with HCV GT4 infection and NS5A polymorphisms who received EBR + GZR with or without RBV achieved SVR12 [33].

#### Daclatasvir

In a Swedish experience, the outcome of IFN-free treatment for HCV relapse after liver transplantation has been documented [34]. In total, 93 patients with HCV relapse after liver transplantation received SOF-based treatment either in combination or not with SMV, DAC or LDV with or without RBV. Of these, 7 patients were infected by GT4 HCV, and all of them achieved SVR12.

Good results were also recorded in the ATU French cohort, composed by 176 GT4 patients treated with SOF and DAC. All the patients enrolled had advanced stages of liver fibrosis and no other treatment option, at the time of the study. The overall SVR 12 rate was 90%, with the highest rate (97%) reached in cirrhotic patients treated with RBV, a the lowest (88%) in those treated without RBV [35].

Babatin et al. reported their real life experience in treating 96 GT4 patients, 56 received SOF + SMV ± RBV (group 1) whereas 40 SOF + DAC ± RBV for 12 weeks (group 2). Liver cirrhosis was present in 53.6 and 35.0% of group 1 and 2, respectively. Furthermore, 43.2% in group 1 and 52.5% in group 2 already failed a previous IFN-treatment. All patients achieved SVR 12 (100% SVR), adverse events were reported in 32% of the patients but none discontinued treatment [36].

### Special populations

#### Renal impairment

A recent multicenter trial investigated the efficacy and safety of OBV/PTV/r and dasabuvir with/without ribavirin in 35 GT1–4 HCV-infected patients undergoing hemodialysis [37]. In this difficult-to-treat population, all patients archived SVR, without any relevant safety concern. The most relevant side effect was anemia, which was more marked in patients on ribavirin (*n* = 17) and requiring increase of the dose of erythropoiesis-stimulating agents. With specific reference to patients with renal impairment Ponziani et al. also reported the outcomes of a series of 10 subjects on hemodialysis therapy infected by GT1a, 1b, or 4 HCV that received OBV/PTV/r (+ DSV in GT1), with or without RBV [37]. All patients achieved SVR12, and therapy was well tolerated.

The OBV/PTV/r regimen does not require dose modification for those with end-stage renal disease, with or without dialysis.

#### End stage liver disease

The efficacy and safety of SOF/LDV association have been also explored in patients with advanced cirrhosis in Child-Pugh stage B or C in an open-label study conducted at 34 sites in Europe [38]. Patients were randomly assigned to 12 or 24 weeks of treatment with SOF/LDV + RBV and were stratified in subgroups on the basis of liver transplantation receipt or not and Child-Pugh stage. Thirty-seven patients with HCV GT4 were enrolled and SVR was achieved in 30 subjects (81%), with higher success rates in the groups treated for 24 weeks.

## Discussion

The wide majority of clinical trials and ‘field-practice’ experiences on HCV treatment are focused on GT1 and GT3, which represent the most frequently-observed genotype worldwide and the most challenging to treat, respectively. Nevertheless, a large proportion of patients is infected by GT4, but this population is often under-represented in clinical trials. Moreover, in the era of peg-IFN and RBV, GT4 was one of the more difficult to treat genotypes, with SVR rates of 43–70% [38]. Therefore, many pre-treated patients, either non-responders or relapsers, are still to be treated in the current era.

On the other hand, data emerging from the analysis of DAA-treated cohorts show an overall SVR rate > 90%, even in the most difficult to cure populations such as patients affected by liver cirrhosis. Available evidence on GT4 therapy is overall scant. However, studies conducted to date – both in the experimental and the ‘field-practice’ setting - have showed promising cure rates up to 100%, even in treatment-experienced or cirrhotic GT4 patients, when administered for 12 weeks courses. Very short treatments of only 6 or 8 weeks seem not yet fully optimized, since also non-cirrhotic naïve patients failed to reach satisfactory percentages of cure in some contexts, even if the number of studied subjects is still low [23]. In such scenario, the desire to spare a few weeks of treatment risks may in turn be associated with high risk of retreatment or onset of resistances. Moreover, all DAAs appear very well tolerated, with discontinuation rates usually below 5%, and side effects often linked more to RBV co-administration than to DAA tolerability issues [27, 32].

Due to the period of analysis of the literature review no more recent combinations have been included. With these combinations the success rates reach 97–99% success. This review is limited in being tightly focused on effectiveness; as such, it does not assess adverse reactions, quality of life, or any other long-term factors [39–41].

The results of this analysis show the pivotal role of OBV/PTV/r, which is characterized by a SVR rate higher than 90% in all categories of patients [17, 27–30] and studied for GT4 also in the context of advanced kidney disease [35, 36]. Likewise SOF/LDV and SMV + SOF achieve high success rates [11, 14–16, 21–26, 28, 34–36]. We must also consider promising results obtained with EBR + GZR [17, 32], glicaprevir/biprentasvir [19, 20, 42] and SOF/VEL in association or not with VOX [8–11]. The data analysed in the present review clearly show that there is not one regimen standing out compared to others, but that there are many regimens that can be safely used and that are effective for GT4 treatment. An approach to GT4 treatment guided by current guidelines is advisable [43–45], with continuous updating of the revolution of the therapeutic scenario currently underway.

## Conclusions

The introduction of DAA therapy clearly revolutionized the approach to treatment of HCV infection. Current evidence shows high SVR rates and an excellent tolerability profile also in patients with GT4 infection, including those with renal impairment. Although larger trials are needed to further confirm these findings, DAAs grant the cure to thousands of patients and give them a new hope to definitively clear HCV GT4 infection, not only in newly infected patients, but also in who underwent to IFN and RBV-related side effects and failed to be cured.
